# Genomic and phenotypic analyses of multidrug-resistant *Acinetobacter baumannii* NCCP 16007 isolated from a patient with a urinary tract infection

**DOI:** 10.1080/21505594.2020.1867421

**Published:** 2020-12-29

**Authors:** Misung Kim, Jaeeun Park, Woojun Park

**Affiliations:** Laboratory of Molecular Environmental Microbiology, Department of Environmental Science and Ecological Engineering, Korea University, Seoul, Republic of Korea

**Keywords:** *Acinetobacter baumannii*, polymyxin B-resistance, whole-genome analysis, horizontal gene transfer, carbapenem-resistance, antibiotic resistance

## Abstract

Polymyxin B (PMB) is increasingly used as a last-line antibiotic; however, the emergence of PMB resistance is a serious threat to global health. Here, a total of 40 *Acinetobacter baumannii* clinical isolates were collected to screen for PMB-resistant strains. Several clinical isolates including NCCP 16007 were far more resistant to PMB (MIC: 128–256 μg/ml) than the ATCC 17978 strain (MIC: 2 μg/ml) and appeared to possess resistance to broad-spectrum antibiotics including meropenem and 12 others. Four highly PMB-resistant strains possessed point mutations in the histidine kinase PmrB, leading to an increased expression of *pmrC* encoding a phosphoethanolamine transferase. Whole-genome analyses revealed that the NCCP 16007 stain had acquired two additional copies of the *pmrC* gene with phage integrase and 13 antibiotic resistance genes (ARGs) from other pathogens, including *Klebsiella pneumoniae* and *Pseudomonas aeruginosa*. The GC ratios of the ARGs (50–60%) were higher than that of the chromosomal backbone (39.06%), further supporting the horizontal gene transfer of ARGs. Comparative genomics with other multidrug-resistant *A. baumannii* strains revealed that the NCCP 16007 strain has many additional ARGs and has lost several virulence factors including Csu pili and heme oxygenase but exhibited high pathogenicity in *Galleria mellonella*-infection models. The observation of condensed biofilm through confocal and scanning electron microscopy suggested that the NCCP 16007 strain may possess high adhesion capacity during urinary tract infection. Therefore, our genomic and phenotypic analyses suggested that the multidrug-resistant *A. baumannii* NCCP 16007 strain possesses high genome plasticity, natural transformation ability, and pathogenicity.

## Introduction

*Acinetobacter baumannii* is a nosocomial pathogen often linked to catheter-related bloodstream infections and ventilator-associated pneumonia infections in immunocompromised patients [1,2]. Due to its ability to develop and spread antibiotic resistance genes (ARGs), the incidence of *A. baumannii* infection in clinical environments has significantly increased in recent years [3]. Multidrug-resistant (i.e. resistant to three or more antimicrobial agents) *A. baumannii* (MRAB) infection is linked to accelerated morbidity and mortality worldwide [4]. Carbapenems and broad-spectrum β-lactam antimicrobial agents including meropenem, imipenem, and doripenem have been considered for the treatment of MRAB [5]. However, a nosocomial outbreak of carbapenem-resistant *A. baumannii* (CRAB) clonal complex 92 (international clone II) with blaOXA-23 encoding a carbapenem-hydrolyzing oxacillinase has been reported in South Korea and many other countries [6–9]. Due to a lack of effective treatment and limited therapeutic options, conventional drugs such as polymyxin B (PMB) and fosfomycin are still used for MRAB and CRAB infections [10,11].

Polymyxin B (PMB) is a cationic and amphipathic therapeutic compound that consists of a cyclic polypeptide linked to a tripeptide linear chain with a fatty acid tail [12]. Although PMB has been linked to some adverse side effects such as neurotoxicity and nephrotoxicity, PMB is still used as a last-line treatment alone or in combination with carbapenem and vancomycin [13]. PMB binds to the lipid A of lipooligosaccharide (LOS) and lipopolysaccharide (LPS) in gram-negative bacteria, which modifies the charge of the negatively charged outer membrane (OM) [14]. LPS is then destabilized, thereby increasing the membrane’s permeability, which causes leakage of components in the cytoplasm and ultimately leads to cell death [15]. Major bacterial PMB resistance occurs when lipid A is modified by the *pmrC*-encoded phosphoethanolamine (PetN) transferase and the *arnT*-encoded 4-amino-4-deoxy-L-arabinose (L-Ara4N) transferase. Both enzymes respectively add positively charged PetN and L-Ara4N to the lipid A, which reduces the binding of PMB to the OM [16,17]. In many PMB-resistant bacteria, several different point mutations found in the *pmrB* gene encoding a histidine kinase might prolong activation of the downstream transcriptional regulator PmrA by phosphorylation of an aspartate residue [18–20]. The phosphorylated PmrA binds to the promoter regions of the *pmrCAB* and *arnBCADTEF* operons, which increases the expression of the PmrC and ArnT enzymes, respectively [21,22]. Activation of the *arnT* operon (*arnBCADTET*, also called as the *pmrHFIJKLM* operon) adds LAra4N groups to lipid A only in several Gram-negative bacteria such as *Escherichia coli, Yersinia pestis, Pseudomonas aeruginosa,* and *Ralstonia solanacearum*, but not in *Citrobacter rodentium* and *A. baumannii* [23,24].

The plasmid-borne *mcr-1* gene, which encodes a PetN transferase, was originally detected in *Escherichia coli* SHP45 isolated from a pig in China [25,26]. The *mcr-1* gene product, which shares a 34% amino acid similarity with the PmrC of *A. baumannii*, might be transferred to other bacteria including *A. baumannii* and *K. pneumoniae* through horizontal gene transfer (HGT) via mobile genetic elements such as insertion sequences (IS), transposons, and conjugative plasmids [27]. IS elements can be classified into different families based on their transposase homologies, target specificity, composition, and length of their terminal inverted repeats [28]. A total of 59 different IS elements wave been identified. In many environmental and clinical *A. baumannii* strains (https://www-is.biotoul.fr/index.php), of which IS*AbaI*, belonging to the IS4 family located upstream of the genes encoding β-lactamase and PetN transferase, appears to be crucial for ARG transfer [19,29]. In this study, the genome of multidrug-resistant *A. baumannii* NCCP 16007 isolated from a patient with a urinary tract infection was analyzed. We hypothesized that *A. baumannii* NCCP 16007 acquired ARGs and IS elements from other bacteria, resulting in variation of GC-content within those regions. Interestingly, two copies of *pmrC* genes with phage integrases were identified, in addition to 13 acquired ARGs. Our genomic analyses suggested that HGT events from other pathogens or plasmids including *K. pneumoniae, P. aeruginosa*, and *Shigella flexneri* were responsible for the multidrug-resistant (MDR) phenotype of the *A. baumannii* NCCP 16007 strain.

## Materials and methods

### Bacterial strains and culture conditions

Forty *A. baumannii* clinical isolates were collected from two different facilities (National Culture Collection for Pathogens, Sungkyunkwan University, South Korea). The bacterial strains used in this study are listed in Table S1. These clinical strains were obtained from 2004 to 2013 in South Korea. The year, region, source, and genotypes of the clinical strains were provided by the National Culture Collection for Pathogens (see our Table 1). The genome of the Lab-WT ATCC 17978 reference strain is available in the NCBI database (Accession number: CP000521). All *A. baumannii* strains studied herein were grown at 37°C in Luria-Bertani (LB) broth with constant shaking at 220 rpm and aeration. All strains were cultured overnight (O/N) for 16 hours, after which each of the O/N cell cultures was diluted 1/100 and cultured for three additional hours to ensure that all experiments were conducted under the mid-exponential phase (optical density at 600 nm: 0.4).

**Table 1.** Antibiotic susceptibility tests of PMB-resistant clinical isolates and *A. baumannii* Lab-WT (ATCC 17978, control)

		MIC (μg/ml) of antibiotics													
Strain	Isolate origin	PMB	COL	AMP	KAM	GEM	TET	RIF	CDM	CTC	DXC	TMP	CRP	STM	MPN
Lab-WT (ATCC 17978)	Laboratory	2	2	256	4	1	1	4	128	1	1	32	128	128	1
**NCCP 16007**	**Urine**	**256**	**> 512**	**> 512**	**> 512**	**> 512**	**512**	**>1024**	**>128**	**32**	**32**	**>128**	**>128**	**>128**	**16**
NCCP 15996	Urine	256	512	> 512	> 512	> 512	256	8	>128	64	32	>128	>128	>128	>32
NCCP 15995	N.A	128	> 512	> 512	> 512	> 512	512	32	>128	32	16	>128	>128	>128	32
F-1629	N.A	128	> 512	> 512	> 512	> 512	512	> 512	>128	32	32	>128	>128	>128	32

Notes: N.A indicates, not available

*PMB, polymyxin B; COL, colistin; AMP, ampicillin; KAM, kanamycin; GEM, gentamicin; TET, tetracycline; RIF, rifampicin; CDM, clindamycin; CTC, chlortetracycline; DXC, doxycycline; TMP, trimethoprim; CRP, chloramphenicol; STM, spectinomycin; MPN, meropenem

### Antimicrobial susceptibility testing

All antibiotics were purchased from Sigma-Aldrich, USA. A total of 14 antibiotics were selected to test the antibiotic resistance of Lab-WT and four PMB-resistant *A. baumannii* strains (NCCP 15995, NCCP 15996, NCCP 16007, and F-1629). The antimicrobial agents used in this study were polymyxin B, colistin, ampicillin, kanamycin, gentamicin, tetracycline, rifampicin, clindamycin, chlortetracycline, doxycycline, trimethoprim, chloramphenicol, spectinomycin, and meropenem. Antimicrobial susceptibility tests were conducted with the broth-dilution method using 96-well plates [30]. The 96-well microtiter plates were incubated at 37°C for 24 hours. After cultivation, optical density (at 600 nm) was measured to determine the minimal inhibitory concentration (MIC). The MICs of PMB were measured in the Lab-WT and the 40 clinical isolates, whereas the MICs of the other 13 antibiotics were determined only in the PMB-resistant *A. baumannii* strains (NCCP 15995, NCCP 15996, NCCP 16007, and F-1629).

### Polymerase chain reaction (PCR) and sequencing of the pmrB gene

The primers used for PCR and sequencing of the *pmrB* gene are specified in Table S2. The PCR was conducted with the following thermal profile: initial denaturation of heating at 95°C for two minutes, 35 cycles at 98°C for 20 seconds, then 60°C for 15 seconds, and 72°C for 30 seconds. The final extension step was conducted at 72°C for five minutes. The PCR products were then cooled to 4°C and sequenced with a 3730xl DNA analyzer (Applied Biosystems, USA).

### Whole-genome sequencing and comparative genomics analysis

Total DNA from O/N cultured *A. baumannii* NCCP 16007 cells was extracted using the FastDNA® Spin Kit for Soil (MP Biomedicals, USA). Whole-genome sequencing was performed using the PacBio sequencing technique (Chunlab, South Korea). The genomic sequences of NCCP 16007 were downloaded from EzGenome (http://ezgenome.ezbiocloud.net/ezg_browse) and analyzed using the CLgenomics software (ChunLab, South Korea). All genomic data were deposited in the NCBI database under the accession number CP049363. Comparative genome analysis was conducted using the ncbi-blast-2.10.0 (https://ftp.ncbi.nlm.nih.gov/blast/executables/blast+/LATEST/) and Mauve software packages (http://darlinglab.org/mauve/download.html).

### Quantitative reverse transcription-PCR (qRT-PCR)

Total RNA of *A. baumannii* was isolated from 5 ml of cell cultures in the mid-exponential growth phase (optical density at 600 nm: 0.4) using the RNeasy Mini Kit (QIAGEN, USA). The qRT-PCR primers used to determine the relative expression of the *pmrC, ompA, bfmR*, and *pilA* genes are listed in Table S2. All procedures were conducted in triplicate from at least three independent cultures. The relative expression levels were compared with the 16s rRNA expression level of each strain.

### ARGs, IS elements, and virulence factor analyses

Acquired ARGs and chromosomal mutations of the Lab-WT and NCCP 16007 strains were detected using the ResFinder software 3.2 (ge.cbs.dtu.dk/services/ResFinder-3.2/), and NCBI BLAST (https://blast.ncbi.nlm.nih.gov/Blast.cgi). The CLgenomics software was then used to identify ARGs in *A. baumannii*. The GC ratio was measured with the GC Content Calculator web tool (https://www.biologicscorp.com/tools/GCContent/#.XnIhjqgzZPY). Insertion sequence (IS) elements and virulence genes were analyzed with the IS finder (https://isfinder.biotoul.fr/blast.php) and VFDB (http://www.mgc.ac.cn/VFs/main.htm) web tools, respectively. The BioProject identifiers (ID) or NCBI accession numbers of the genomes used herein were PRJNA609015 (NCCP 16007), RJNA42153 (1656-2), PRJNA21111 (AB0057), PRJNA17827 (ACICU), PRJNA487603 (UPAB1), NC_011595 (AB307-294), PRJNA28921 (AYE), PRJNA74421 (BJAB07104), PRJNA74423 (BJAB0715), PRJNA74425 (BJAB0868), PRJNA61919 (D1279779), PRJNA52959 (MDR-TJ), PRJNA28333 (MDR-ZJ06), PRJNA13001 (SDF), NC_017387 (TCDT-AB0715), and PRJNA74551 (TYTH-1).

### Galleria mellonella-infection model

*Galleria mellonella* larvae were obtained from SWORM, South Korea. Prior to injection, healthy *G. mellonella* larvae were starved to increase susceptibility to infection by suppressing the immune response for 4 h at 20°C. The larvae were then placed on ice and injected in the last left proleg with 10 µl of 106 CFU of either Lab-WT or NCCP 16007 strain with-/-without PMB (1/2 MIC; 1 μg/ml for Lab-WT, 128 μg/ml for NCCP 16007) using 31-gauge 6 mm syringes (BD, USA). A total of 15 *G. mellonella* larvae per condition were then tested after inoculation. The larvae were incubated in Petri dishes (SPL Life Sciences, South Korea) containing 100 mg of wheat bran powder (MG Natural, South Korea) at 37°C and inspected and scored every 24 h for death, failure to move in response to touch, and melanization.

### Measurement of biofilm formation

Biofilms were grown in confocal dishes (SPL Life Sciences, South Korea) at 37°C for 24 h in LB broth. The biofilm cells were stained with FilmTracer™ SYPRO Ruby (Invitrogen, USA) for 30 min at room temperature, protected from light, and then washed with distilled water. The observed confocal laser scanning microscopy (CLSM; Carl Zeiss, Germany) images were analyzed and modified using the Zen 2.1 (Blue edition; Carl Zeiss, Germany) software.

LB broth in sterile 96-well microtiter plates (SPL Life Sciences, South Korea) was inoculated in triplicate with each overnight LB-grown culture and diluted 1:100 in LB broth. The volume of the cells was determined by converting the OD600 value of the O/N cells. Uninoculated LB broth was used as a negative control. The microtiter plates were then incubated at 37°C for 24 h. After removing planktonic cells, the biofilm biomass was stained with crystal violet and solubilized with 95% ethanol (v/v), after which its absorbance was measured at 550 nm.

### Field-emission scanning electron microscopy (FE-SEM) analysis

Mid-exponential growth phase NCCP 16007 strain cells were used for SEM analyses. The cells were first fixed with low-strength Karnovsky’s solution (2% paraformaldehyde, 2.5% glutaraldehyde, and 0.1 M phosphate buffer, final pH 7.2) for 4 h. Secondary fixation was done using 2% osmium tetroxide solution at 4°C for 2 h. These fixed samples were gradually dehydrated with ethanol (30%, 50%, 70%, 80%, 90%, 100%) for 10 min each and placed on an aluminum stub overnight to dry at room temperature. These samples were then coated with platinum and analyzed using a field-emission scanning electron microscope (Quanta 250 FEG; FEI, USA).

### Esterase activity assay using 4-nitrophenyl acetate

4-nitrophenyl acetate, the substrate used in esterase activity assay, was first dissolved in methanol (10 mg/ml) (Sigma-Aldrich, USA). An aliquot (40 μl) of the cell cultures was then mixed with 120 μl phosphate buffer at pH 7.2 and 40 μl of the substrate within 96-well microtiter plates. After incubation at 37°C for 15 min, the mixtures were analyzed at 405 nm, following the hydrolysis of 4-nitrophenyl acetate. Distilled water was used as a negative control.

## Results

### MDR of PMB-resistant clinical isolates

A total of 40 *A. baumannii* clinical isolates were obtained from sputum (25%), pus (17.5%), and urine (12.5%) samples; the source of the 55% remaining isolates was unknown (Table S1). The sources and genotypes of the bacterial isolates were provided by the National Culture Collection for Pathogens in South Korea (http://nccp.cdc.go.kr/main.do?menu_id=010100). These 40 clinical isolates included 25 (62.5%) strains from clonal complex (CC) 92 (international clone II), all of which have been confirmed to carry a *blaOXA-23* gene encoding a carbapenem-hydrolyzing class D β-lactamase (Table S1) [7,8]. Interestingly, two strains (NCCP 15994 and NCCP 15998) featured a *blaOXA-51* gene downstream of the IS*AbaI* gene (Table S1). The PMB resistance of our clinical isolates was tested to determine the MIC via the liquid dilution method [30]. Eight of the strains (20%, 8/40) had higher MICs (4 ~ 256 μg/ml) than the wild-type ATCC 17978 strain (MIC, 2 μg/ml) (Table S1). Moreover, four PMB-resistant strains exhibited much higher resistance profiles compared to all other 13 tested antibiotics, including colistin and meropenem relative to the Lab-WT (ATCC 17978) strain (Table 1). Three of the PMB-resistant strains were genotyped and found to harbor the *blaOXA-23* gene had a 16- to 32-fold higher MIC (16 ~ 32 μg/ml) to meropenem than the WT (1 μg/ml) (Table 1 and Table S1).

**Figure 1.**
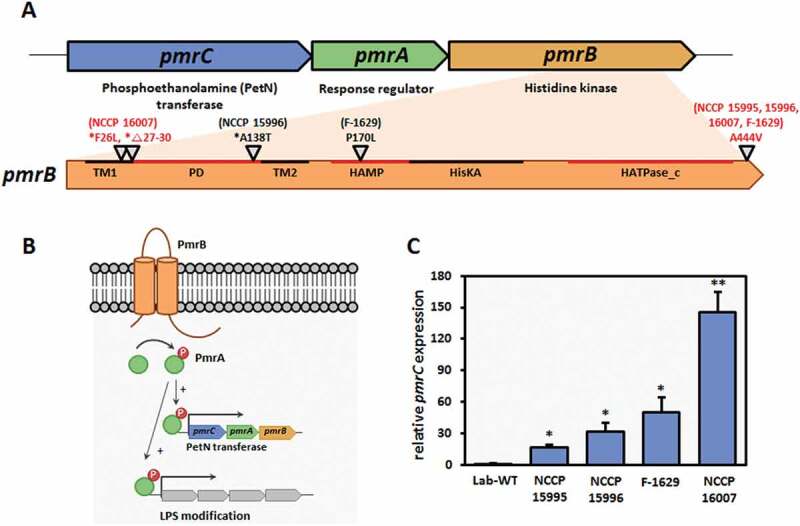
PmrAB-mediated PMB resistance and *pmrC* expression levels. (a) Genomic organization of the *pmrCAB* operon. *pmrB* mutations in the highly PMB-resistant clinical isolates. PmrB domains: TM1, first transmembrane domain (aa 10–29); PD, periplasmic domain (aa 29–142); TM2, second transmembrane domain (aa 142–164); HAMP, histidine kinases, adenylylcyclases, methyl-binding proteins, and phosphatases (aa 145–214); and HATPase_c, histidine kinase-like ATPases (aa 326–437) [10]. The red letters indicate mutation locations in the NCCP 16007 strain. * novel site mutation in the PmrB. (b) Schematic representation of PMB resistance mechanism in *Acinetobacter baumannii*. (c) High expression of the *pmrC* gene in highly PMB-resistant clinical isolates (NCCP 15,995, NCCP 15,996, F-1629, and NCCP 16007 strains). The *pmrC* expression of the strains was normalized to their respective 16S rRNA expression. Data represent the mean (± standard deviation, SD; N = 3 biological replicates). *, *P* < 0.05; **, *P* < 0.01 by Student’s *t*-test against theoretical value

Modulation of the OM charge by the PmrA/B two-component system-activated PmrC appeared to confer PMB resistance to pathogenic *A. baumannii* strains [14]. It was speculated that a mutation in the *pmrB* gene leads to higher expression of the downstream *pmrC* gene, which might lead to higher PMB MICs in the four strains that exhibited this phenotype [18–20]. DNA analysis of PCR products with the *pmrB*-specific primer pairs indicated the occurrence of either point mutations or deletion in our tested strains (Figure 1(a) and Table S2). Only the NCCP 16007 strain had four amino acid deletions (Δ27-30) in the first transmembrane (TM) region. All four PMB-resistant strains exhibited the same point mutation in PmrB (A444V) and carried different point mutations in distinct regions (novel F26L in TM, NCCP 16007; novel A138T in the periplasmic domain, NCCP 15996; and P170L in HAMP domain, F-1629). Although the mechanisms by which these mutations affect PmrA-phosphorylation remain unclear, our qRT-PCR analysis showed that the four tested strains had 20- to 150-fold higher *pmrC* expression levels compared to the Lab-WT strain (Figure 1(c)). Notably, given that the NCCP 16007 strain possesses the MDR phenotype, the highest PMB MIC, and the highest level of *pmrC* expression (150-fold increase), its genome should be investigated to dissect the detailed mechanisms of PMB resistance and MDR phenotypes.

### Whole-genome analysis of multidrug-resistant A. baumannii NCCP 16007

The genome of NCCP 16007 was assembled into three contigs 3,884,786 bp in length with 3,618 coding regions, consisting of one chromosomal backbone (3,854,994 bp) and the pNC1 (8,031 bp) and pNC2 (21,761 bp) plasmids (Table S3). When compared to the average length of the 4,536 *A. baumannii* genomes (3,974,730 bp) listed in the NCBI database, the chromosomal length of NCCP 16007 is 120 kb shorter and its GC content (39%) is consistent with those of other *A. baumannii* strains. The NCCP 16007 strain whose genome size is shorter than the Lab-WT strain (ATCC 17978, 3,976,747 bp), lacked genes associated with cytosine-specific DNA methyltransferase (e.g. *dcm*), type IV secretion (e.g., *trbL* and *tadA*), and colicin V secretion (e.g. *cvaA* and *cvaB*), as well as other functionally unknown genes. The NCCP 16007 strain had the same 16S rRNA as ATCC 17978 but displayed a different average nucleotide identify (ANI) value (97.74%) from the Lab-WT strain, suggesting that many of its genes might have been horizontally transferred from other bacteria (Figure S1). Unlike two of the plasmids present in the Lab-WT strain (pAB1: 13,408 bp; pAB2: 11,302 bp), the plasmids of the NCCP 16007 strain were distinctively characterized by their size and presence of genes encoding a TonB-dependent receptor and a phage integrase (Table S3).

Interestingly, the NCCP 16007 strain harbors a total of three copies of the *pmrC* gene that are 97% identical to each other, whereas the Lab-WT possesses only one *pmrCAB* operon. The *pmrC* and *eptA* genes have been reported as the same functional genes encoding PetN transferases [23,31]. The chromosome contains a *pmrC* gene in the *pmrCAB* operon and two *pmrC* gene copies are located next to the phage integrase genes in both the chromosome and pNC1 plasmid (Table 2). The 150-fold increase in *pmrC* expression observed in this strain might be due to its location next to the IS element, which contains a strong promoter sequence [32,33] and 3 copies of the *pmrC* gene with 98% homology with the Lab-WT at the gene level (Figure 1(c) and Table S2). Our genome analyses therefore suggest that both the mutation of the *pmrB* gene and the presence of three *pmrC* gene copies resulted in high levels of *pmrC* expression and consequently high PMB MIC values among all tested strains (Table 1).

**Table 2.** The origins of ARGs in multidrug-resistant *A. baumannii* NCCP 16007

Antibiotics	AR gene	Homology to source (%)	Query/Template length (bp)	Position in contig	Product	Souce	GC content of AR gene/souce (%)		Accession number
Macrolide	*mph(E)*	100	885/885	1,257,484–1,258,368	Macrolide 2ʹ-phosphotransferase	Uncultured bacterium plasmid pRSB105	36	56.7	DQ839391
	*msr(E)*	100	1476/1476	1,255,953–1,257,428	ABC-F type ribosomal protection protein	*Pasteurella multocida*	39	40	FR751518
	*macB*	98	1959/1995	579,699–581,693	Macrolide export ATP-binding/permease protein	*Acinetobacter baumannii* ATCC 17978	45	39	CP000521
	*macA*	99	1326/1341	581,696–583,036	Macrolide export protein	*Acinetobacter baumannii* ATCC 17978	40	39	CP000521
Quinolone, Fluoroquinolone	*aac(6ʹ)-Ib-cr*	100	519/519	1,246,100–1,246,618	Aminoglycoside acetyltransferase	*Klebsiella pneumoniae* strain M7943	54	46	EF636461
	*gyrA*	98	2698/2715	2,951,939–2,954,653	Type Ⅱ topoisomerase	*Acinetobacter baumannii* ATCC 17978	42	39	CP000521
Sulfonamide	*sul1*	100	840/840	1,248,697–1,249,536	Dihydropteroate synthase	*Pseudomonas aeruginosa* plasmid R1033	61	59	U12338
Aminoglycoside	*aac(6ʹ)-Ib3*	100	555/555	1,246,064–1,246,618	Aminoglycoside 6ʹ-N-acetyltransferase	*Pseudomonas aeruginosa* plasmid pCFF04	53	54	X60321
	*aadA1*	100	792/792	1,247,401–1,248,192	Streptomycin 3”-adenylyltransferase	*Pseudomonas aeruginosa* K7	52	66	JQ414041
	*armA*	100	774/774	1,252,881–1,253,654	Aminoglycoside methyltransferase	*Klebsiella pneumoniae* strain BM4536 plasmid plP1204	29	46	AY220558
	*aph(6)-ld*	100	837/837	244,530–245,366	Streptomycin phosphotransferase	*Escherichia coli* plasmid RSF1010	53	61	M28829
	*aph(3”)-lb*	100	804/804	245,366–246,168	Aminoglycoside phosphotransferase	*Pseudomonas aeruginosa*	54	66	AF024602
	*aadA*	96	428/447	2,402,465–2,402,911	Streptomycin 3”-adenylyltransferase	*Acinetobacter baumannii* ATCC 17978	44	39	CP000521
	*aadA*	100	3441/3441	2,489,804–2,493,244	Streptomycin 3”-adenylyltransferase	*Acinetobacter baumannii* strain N13-03449	52	39	CP043417
	*aac2-Ⅰ*	99	537/543	191,557–192,099	Gentamicin 2ʹ-N-acetyltransferase	*Acinetobacter baumannii* ATCC 17978	35	39	CP000521
β-lactam	*blaADC-25*	100	1152/1152	2,697,522–2,698,673	β -lactamase	*Acinetobacter baumannii* strain 17,368	36	39	EF016355
	*blaOXA-23*	100	822/822	234,131–234,952	β -lactamase	*Acinetobacter baumannii* strain DR25547/96	36	39	AY795964
	*blaOXA-66*	100	825/825	2,049,430–2,050,254	β -lactamase	*Acinetobacter baumannii*	38	39	AY750909
	*blaTEM-1D*	100	861/861	2,493,492–2,494,352	β -lactamase	*Escherichia coli*	48	51	AF188200
	*hcpA*	100	564/564	3,594,869–3,595,432	β -lactamase	*Acinetobacter baumannii* ABCR01	36	39	CP042931
	*penP*	97	1206/1248	2,365,890–2,367,134	β -lactamase	*Acinetobacter baumannii* ATCC 17978	37	39	CP000521
Carbapenem	*oprD*	82	636/771	216,167–217,483	Outer membrane porin	*Acinetobacter baumannii* ATCC 17978	40	40	CP000521
Phenicol	*catB8*	100	633/633	1,246,711–1,247,343	Chloramphenicol O-acetyltransferase	*Klebsiella pneumoniae*	46	57	AF227506
Tetracycline	*tet(B)*	100	1200/1206	239,958–241,157	Tetracycline efflux pump	*Shigella flexneri* 2b plasmid R100	42	52	AP000342
	*tet(R)*	100	624/624	241,239–241,862	Transcriptional repressor	*Shigella flexneri* 2b plasmid R100	39	52	AP000342
Rifampicin	*rpoB*	99	4068/4089	310,545–314,633	β subunit of bacterial RNA polymerase	*Acinetobacter baumannii* ATCC 17978	42	39	CP000521
Polymyxin B	*pmrC-3*	98	1368/1390	pNC1: 1593–2828	Lipid A phosphoethanolamine transferase	*Acinetobacter baumannii* ATCC 17978	35	39	CP000521
	*pmrC-2*	99	1176/1193	1,230,214–1,231,860	Lipid A phosphoethanolamine transferase	*Acinetobacter baumannii* ATCC 17978	37	39	CP000521
	*pmrC-1*	99	1580/1602	3,117,643–3,119,244	Lipid A phosphoethanolamine transferase	*Acinetobacter baumannii* ATCC 17978	37	39	CP000521
	*pmrA*	99	671/675	3,116,954–3,117,628	Response regulator	*Acinetobacter baumannii* ATCC 17978	37	39	CP000521
	*pmrB*	99	1320/1335	3,115,606–3,116,928	Histidine kinase	*Acinetobacter baumannii* ATCC 17978	38	39	CP000521
Multidrug	*qacE*	100	115/115	1,248,356–1,248,703	Multidrug efflux protein	*Salmonella enterica* R17.5425	50	52	WP_000302056

### Acquired antibiotic resistance genes and insertion sequence elements

**Figure 2.**
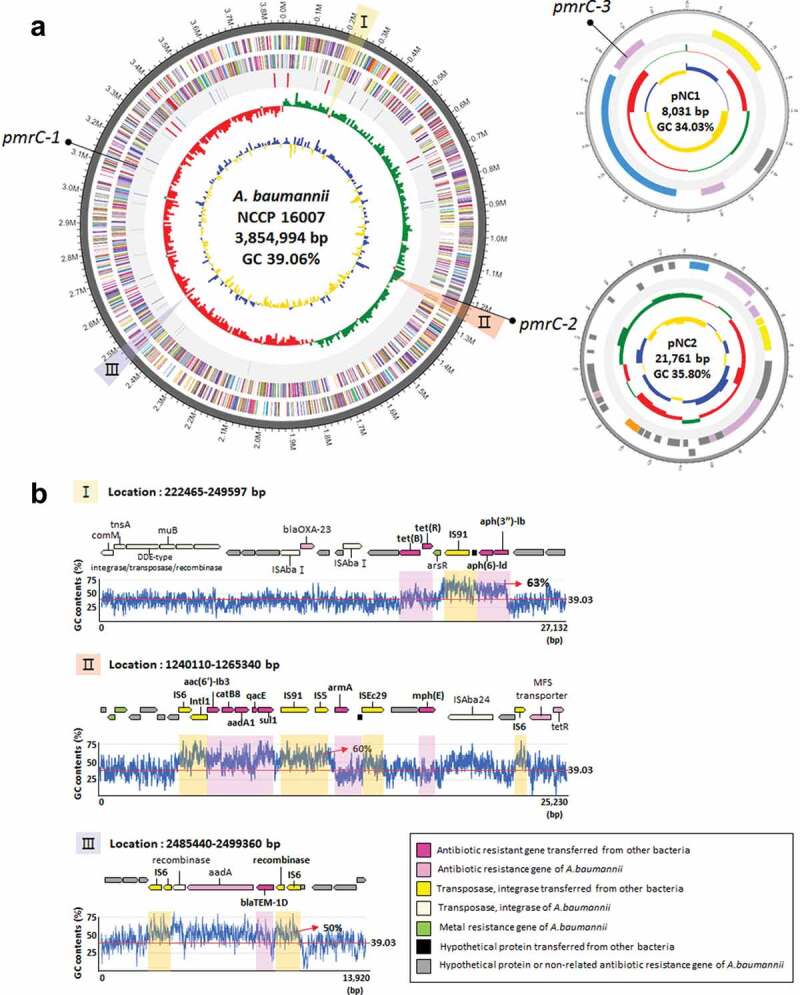
Genetic map and antibiotic resistance islands of the NCCP 16007 strain. (a) Chromosomal backbone and pNC1 and pNC2 plasmids of the NCCP 16007 strain. The circles represent (from outside to inside) forward CDS, reverse CDS, rRNA and tRNA, GC skew (green: +/red: -) and GC ratio (blue: above average value/yellow: below average value). (b) Three antibiotic resistance island sites and their GC contents. The red lines indicate chromosomal GC ratios in the NCCP 16007 strain. Each yellow and pink box represents IS elements and the ARGs transferred from other pathogens

Our genome analyses demonstrated that the NCCP 16007 strain possessed a total of 32 ARGs linked to six different classes of antibiotics including carbapenem, β-lactam, gentamicin, and macrolide (Table 2). Several ARGs in the genome of the NCCP 16007 strain could have contributed to the MDR phenotype against all 14 antibiotics tested (Table 1). The GC ratios (50–60%) of many ARGs including *sul1, aac (6ʹ)-lb3, aadA1, aph (3”)-lb*, and *blaTEM-1D* were much higher than that of the chromosomal backbone (39.06%), leading us to speculate that the ARGs may have been acquired from other bacterial species through HGT (Figure 2). Therefore, we analyzed the homology of the ARGs and IS elements to determine whether the ARGs originated from other bacterial species using the Center for Genomic Epidemiology database and IS finder (see Materials and Methods section, Figure 2(b), Table 2, and Table S4). Fourteen of the 32 ARGs (including *qacE*, which encodes the efflux pump of *Salmonella* sp.) appeared to be originated from other pathogens, with gene homologies exceeding 98% identities (Table 2). The similarity between the GC ratio of the original bacteria and that of each ARG further suggested that the NCCP 16007 strain acquired the genes from clinical environments (Table 2).

Interestingly, there are a total of five different aminoglycoside resistance genes in the NCCP 16007 genome, which make cells remarkably resistant to many aminoglycoside antibiotics (100–500-fold, Table 1). Three genes (*aac (6ʹ)-lb3, aadA1*, and *aph (3”)-lb*) were likely transferred from *P. aeruginosa*, one *armA* gene from *K. pneumoniae*, and one *aph (6)-ld* gene from *E. coli* (Figure 2(b) and Table 2). Additionally, a total of 19 different IS elements next to the ARGs might originate from many different pathogens including *Proteus vulgaris* and *K. pneumoniae* (Table S4). One IS91 family element was found to be identical (100%) to the ISVsa3 of *Vibrio salmonicida*, which is adjacent to the *tet(R)* and the *aph(6)-ld* genes (Figure 2(b–I) and Table 2). Several different IS elements (IS91, IS26, IS5, and Intl1) with high sequence identities (>99%) with those of other pathogenic bacteria were also present near the *sul1, qacE, aadA1, catB8*, and *aac (6ʹ)-lb3* genes (Figure 2(b-II) and Table 2). The *blaTEM-1D* gene encoding a β-lactamase from *E. coli* was located between two IS6 family elements (Figure 2b-III). Unlike the single copy of the IS*AbaI* gene that was adjacent to the *sul2* gene encoding a dihydropteroate synthases in the Lab-WT (Table S5), a total of 15 copies of the IS*AbaI* gene were scattered across the chromosome without the *sul2* gene in the NCCP 16007. Several IS*AbaI* genes are located near the *blaADC* and *blaOXA-23* genes (Table S4). Therefore, the extraordinary MDR capacity of the NCCP 16007 strain against the 14 antibiotics tested herein was due to the large numbers of ARGs and IS elements in its genome.

**Figure 3.**
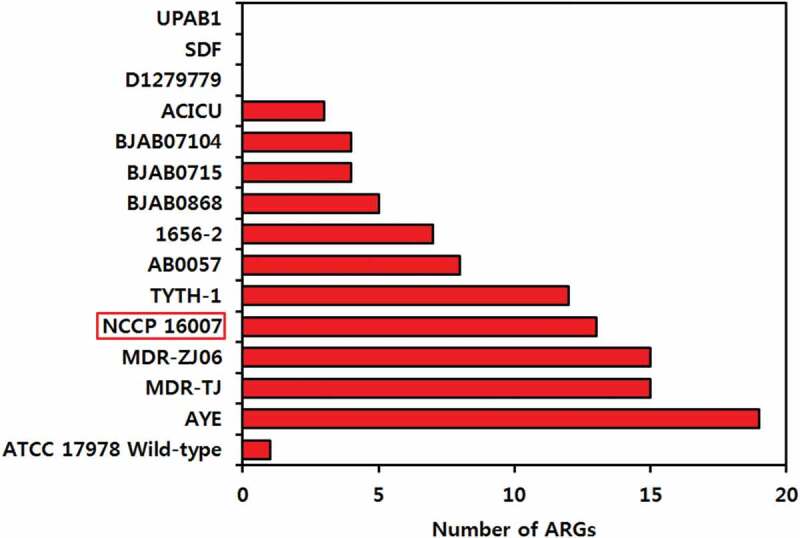
The strains tested herein (Lab-WT and NCCP 16007 stains), as well as 13 other well-known clinical isolates, were investigated to identify acquired ARGs using the Center for Genomic Epidemiology database. Only ARGs acquired from other bacteria were considered, and naturally-occurring ARGs in *Acinetobacter baumannii* were excluded

The genomes of the strains tested herein (Lab-WT and NCCP 16007) as well as 13 other well-known clinical isolates were investigated to identify ARGs using the Center for Genomic Epidemiology database and the Mauve software (Figure 3 and Figure S2). Interestingly, the *A. baumannii* 1656–2 and ACICU strains, which were respectively isolated from the sputum of a hospitalized patient in South Korea and the cerebrospinal fluid of a patient in Italy, also exhibited multiple copies of the *pmrC* gene (Figure S2). The AYE strain also possessed a total of 19 commonly acquired ARGs, and unlike the other studied strains, the AYE strain obtained the trimethoprim resistance genes *dfrA1* and *dfrA10* from *E. coli* (Figure 3 and Figure S2). In contrast to the uropathogenic UPAB1 strain, which does not possess acquired ARGs, the NCCP 16007 strain isolated from a urinary tract infection patient had a total of 13 ARGs (except for the *qacE* efflux pump gene), a large number compared to 13 other clinical strains (Figure 3).

### High pathogenicity in Galleria mellonella-infection models

**Figure 4.**
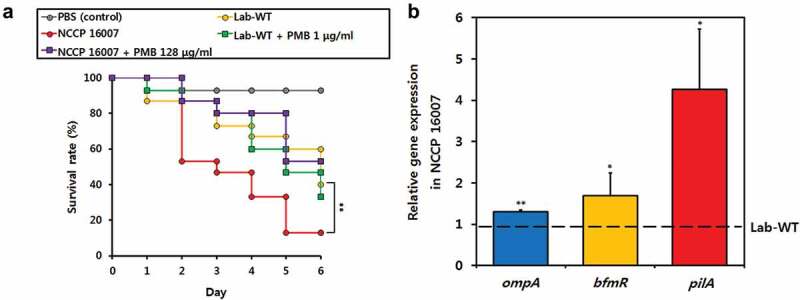
Pathogenicity of the Lab-WT and NCCP 16007 strains. (a) A total of 15 *Galleria mellonella* larvae per condition were infected with the Lab-WT and NCCP 16007 strains to compare their pathogenicity *in vivo*. 10^6^ CFU of the Lab-WT or NCCP 16007 strains coupled with 1/2 of the PMB MICs (1 μg/ml for Lab-WT, 128 μg/ml for NCCP 16007) were mixed and injected into the larvae with a syringe. Mortality was defined as a lack of response or melanization for six days in infected *G. mellonella* at 37°C. Data represent the mean (± standard deviation, SD; N = 15 biological replicates). **, *P* < 0.01 by Student’s *t*-test against theoretical value. (b) The expression of the *ompA, bfmR*, and *pilA* genes was quantified via qRT-PCR. The gene expression profiles of the strains was normalized to their respective 16S rRNA expression. Data represent the mean (± standard deviation, SD; N = 4–6 biological replicates). *, *P* < 0.05; **, *P* < 0.01 by Student’s *t*-test against theoretical value

The virulence factor genes of the NCCP 16007 strain were compared with those of the Lab-WT, other MRAB, and antibiotic-sensitive *A. baumannii* strains (Figure S3). The NCCP 16007 strain appeared to have fewer virulence factors (76%, 44 out of 58 genes) and lacked several important genes that encode Csu pili and heme oxygenase, whereas the known MRAB strains *A. baumannii* TCDT-AB0715 and TYTH-1 exhibited many virulence-related genes (98%, 57 out of 58 genes) (Figure S3). However, a *Galleria mellonella*-infection study revealed that the NCCP 16007 strain was highly pathogenic *in vivo* (Figure 4(a)). A total of 15 *G. mellonella* larvae were tested per condition after inoculation with 10^6^ CFU of either the Lab-WT or NCCP 16007 strains with-/-without PMB (1/2 MIC; 1 μg/ml for Lab-WT, 128 μg/ml for NCCP 16007). The larvae exposed to the NCCP 16007 strain displayed a 90% mortality rate within five days, whereas only a 65% mortality rate was observed in the Lab-WT-exposed larvae (Figure 4(a)). PMB treatment (1/2 MIC) reduced the larvae death rates from 90% to 45% in the NCCP 16007 infection samples; however, the PMB has no effect on infection outcome for the sensitive WT strain probably due to low *in vivo* PMB (1/2 MIC) efficacy against WT strain (Figure 4(a)).

**Figure 5.**
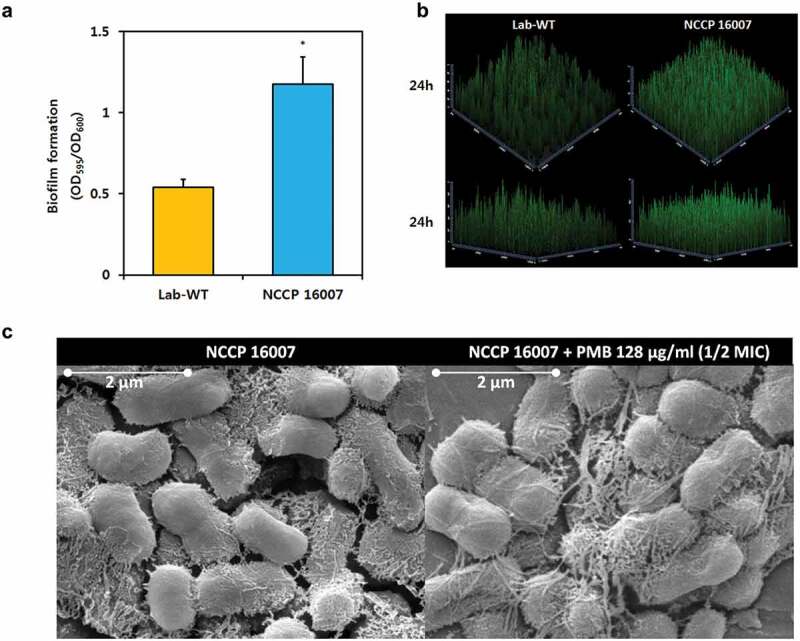
Biofilm formation of the NCCP 16007 strain. (a) Crystal violet quantification of biofilm formation in the Lab-WT and NCCP 16007 strains. (b) Confocal laser scanning microscopy (CLSM) images of biofilms in the Lab-WT and NCCP 16007 strains. Microscopic analysis was conducted using the Film Tracer^TM^ Sypro Ruby dye at 24 h. (c) SEM images of the NCCP 16007 strain. Pili-like biofilm structures were observed in the PMB-treated and non-treated samples. Bacterial cell samples were fixed, washed, dehydrated, dried, and coated with platinum prior to imaging. Data represent the mean (± standard deviation, SD; N = 3 biological replicates). *, *P* < 0.05 by Student’s *t*-test compared to theoretical value

We assumed that although the NCCP 16007 strain has fewer virulence factors than Lab-WT and other clinical strains, the high mortality rate in the larvae infection study would be due to the high expression of pathogenicity-associated genes. The relative expressions of the outer membrane protein, *ompA*, the response regulator of biofilm formation, *bfmR*, and the main subunit of pili, *pilA* genes were respectively 1.3-fold, 1.5-fold, and fourfold higher than those in the Lab-WT, confirming the high virulence of the NCCP 16007 strain (Figure 4(b)). Additionally, virulence-associated biofilm formation was measured in the Lab-WT and NCCP 16007 strains (Figure 5). The crystal violet assay for biofilm quantification indicated that the NCCP 16007 strain produced twofold more biofilm than the Lab-WT strain (Figure 5(a)). Interestingly, unlike the Lab-WT strain, the NCCP 16007 strain formed thick, condensed, and pili-like biofilms within 24 h (Figure 5(b, c)). The PMB-treated NCCP 16007 strain at 1/2 MIC (128 μg/ml) produced longer pili-like biofilms than the untreated samples (Figure 5(c)). Moreover, a twofold increase in esterase activity compared to the Lab-WT strain suggested that the NCCP 16007 strain might lead to more invasive infections and dissemination in the host cells (Figure S4). Therefore, the high mortality and biofilm production of the NCCP 16007 strain may translate to more severe infectious diseases in clinical settings.

## Discussion

**Figure 6.**
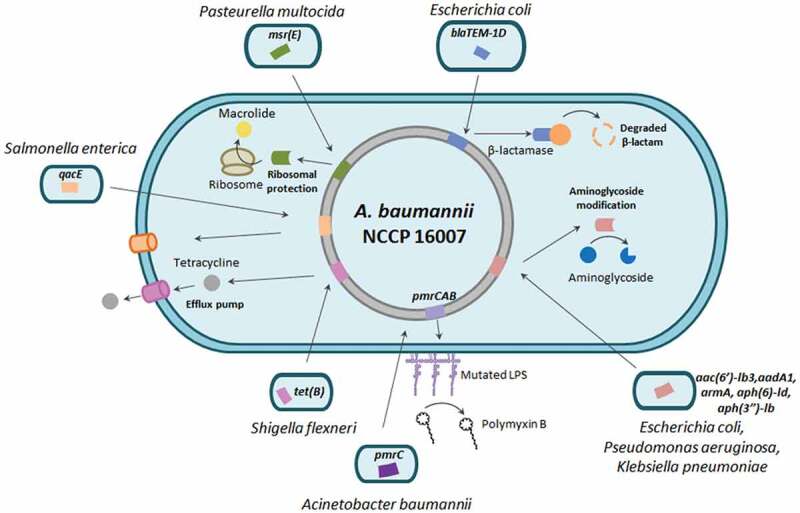
Diagram of multidrug resistance mechanisms in *A. baumannii* NCCP 16007. The β-lactamase (*blaTEM-1D*) potentially transferred from *E. coli* decomposes the β-lactam ring through hydrolysis. Acquired genetic determinants including *aadA1* and *aph(6)-ld* express aminoglycoside transferases and modify gentamicin, kanamycin, and spectinomycin, leading to cell growth arrest. The Msr(E) transferred from *P. multocida* binds to the exit site of the ribosome and protects the ribosome from macrolides. An efflux pump (Tet(B)) acquired from *S. flexneri* actively eliminates tetracycline and chlortetracycline from the cell. Two additional copies of *pmrC*-encoded PetN transferase modulate the charge of lipid A and inhibit the binding of PMB to the OM

Pathogenic bacteria possess a variety of molecular strategies to survive under antibiotic selective pressure. These strategies include random mutations, acquisition of ARGs through HGT, and activation of mobile DNA elements [34]. *Acinetobacter* species have a high genome plasticity and natural transformation ability, which could allow them to evolve by exchanging ARGs under antibiotic conditions [35]. *Acinetobacter baylyi* ADP1 has been reported to possess a remarkable natural competence ability, which is more than 100-fold higher than that of *E. coli* cells treated with CaCl2 [36]. Surprisingly, the MDR *A. baumannii* NCCP 16007 strain likely acquired many ARGs and IS elements through HGT, which resulted in high levels of resistance to most of the tested antibiotics (Table 2 and Table S4). The potential MDR mechanisms in the NCCP 16007 strain are summarized in Figure 6.

The β-lactamases that inhibit β-lactam antibiotics (e.g. ampicillin and meropenem) can be divided into four types (classes A, B, C, and D) based on sequence similarities [37]. The *blaTEM-1D* gene product, which was first discovered in *E. coli*, belongs to the plasmid-encoded constitutive broad-spectrum class A β-lactamases (Table 2 and Figure 6) [38]. The NCCP 16007 strain possessed resistance to ampicillin (MIC > 512 μg/ml) and meropenem (MIC, 16 μg/ml, Table 1) not only due to a mutation in the *oprD* gene (Table 2 and Figure 6) but also due to the presence of several β-lactamases, including the class D β-lactamase (*blaOXA-23* and *blaOXA-66* genes) and class A β-lactamase genes (GC ratio 48%) derived from *E. coli* (*blaTEM-1D*). Bacterial survival strategies against aminoglycoside antibiotics include i) inactivation of aminoglycosides by modifying enzymes, ii) enhanced production of efflux pumps, iii) reduction of membrane permeability, and iv) interference with aminoglycoside binding via modification of the 16S ribosomal RNA [39]. The NCCP 16007 strain possesses several HGT-mediated aminoglycoside-resistance genes from *E. coli* (*aph(6)-ld*, GC ratio 53%), *P. aeruginosa* (*aac(6ʹ)-lb3, aadA1*, and *aph(3”)-lb*, GC ratio 53%), and *K. pneumoniae* (*armA*, GC ratio 29%), which could modify aminoglycosides by adding phosphate, acetyl, adenyl, or methyl groups (Figure 6 and Table 2). Macrolides with a macrocyclic lactone ring (e.g. erythromycin and clarithromycin) bind a 50S ribosome subunit with a specific target in the 23S rRNA and diverse ribosomal proteins [40]. Moreover, the *msr(E)* gene in the genome of the NCCP 16007 strain, which originated via HGT from *P. multocida* (GC ratio 39%), could protect ribosomes from macrolides [41] (Figure 6 and Table 2).

PmrAB-mediated lipid A modification participates in PMB resistance by changing membrane stability [42]. In the *Enterobacteriaceae, P. aeruginosa*, and *A. baumannii*, the PetN transferase PmrC, regulated by the PmrAB two-component system, reduces PMB affinity to LPS [19]. In our study, four PMB-resistant *A. baumannii* clinical isolates exhibited point mutations in the *pmrB* gene and high *pmrC* expression (Figure 1). It is not clear to what extent did the mutations in the *pmrB* affected the expression of *pmrC* and which PmrB domain was predominantly involved in PMB resistance. However, the *pmrC* gene expression of the four strains, 20- to 150-fold higher than that of the Lab-WT strain, supported the hypothesis that lipid A modifications would prevent PMB from binding to the OM (Figure 1).

Many uropathogens initiate a urinary tract infection (UTI) using surface appendages, capsular polysaccharides, adhesins, and pili, which form biofilms by promoting bacteria-bacteria interactions [43,44]. A UTI is generally caused by contamination around the urethra by uropathogens in the intestine, followed by the colonization of the urethra and subsequent mobilization of the pathogens into the bladder, requiring appendages such as pili [45]. In this study, *A. baumannii* NCCP 16007 isolated from a patient with UTI also formed pili-like biofilms and the high expression of *pilA* encoding the type IV pili major subunit was determined (Figures 4 and Figures 5). Paradoxically, the NCCP 16007 strain does not possess the Csu pili-related genes (*csuA, csuB, csuC, csuD*, and *csuE*) needed to form biofilms, but the expression of the top regulator *bfmR* was 1.5-fold higher than that of the Lab-WT strain (Figure S3 and Figure 4). These results indicate that Csu pili may not be essential for biofilm formation or that they could be replaced by other pili systems such as type IV pili [46].

In conclusion, four of the 40 strains of clinical strains isolated in South Korea were highly resistant to PMB, of which the genomic and phenotypic characteristics of NCCP 16007 with the highest *pmrC* expression were analyzed. We assumed that the NCCP 16007 strain has high resistance to all tested 14 antibiotics due to the HGT-mediated ARGs from other pathogen and plasmids. Interestingly, genomic analysis revealed that the NCCP 16007 uropathogenic strain acquired a total of 13 ARGs from other pathogens, a large number of ARGs compared with other clinical strains. Phenotypic analyses revealed that the NCCP 16007 strain exhibited an MDR phenotype, higher pathogenicity, and superior biofilm structure compared with the wild-type. Therefore, *A. baumannii* NCCP 16007 possesses a remarkable repertoire of MDR strategies for PMB resistance, including a large array of horizontally transmitted ARGs and extremely high levels of *pmrC* gene expression.

## Supplementary Material

Supplemental Material
