# Supplementary material for: Genomic and phenotypic analyses of multidrug-resistant Acinetobacter baumannii NCCP 16007 isolated from a patient with a urinary tract infection
Source: Virulence. 2020 Dec 29;12(1):150–64. doi: 10.1080/21505594.2020.1867421 (PMC7781626; doi:10.1080/21505594.2020.1867421)
Supplement: Supplemental Material [file KVIR_A_1867421_SM5889.docx]

**Genomic and phenotypic analyses of multidrug-resistant *Acinetobacter baumannii* NCCP 16007 isolated from a patient with a urinary tract infection**

Misung Kim, Jaeeun Park, and Woojun Park*

*Laboratory of Molecular Environmental Microbiology, Department of Environmental Science and Ecological Engineering, Korea University, Seoul 02841, Republic of Korea*

^*^Corresponding author: Dr. Woojun Park, Department of Environmental Science and Ecological Engineering, Korea University, Seoul 02841, Republic of Korea

E-mail: wpark@korea.ac.kr

Fax: +82-2-953-0737

Phone: +82-2-3290-3067


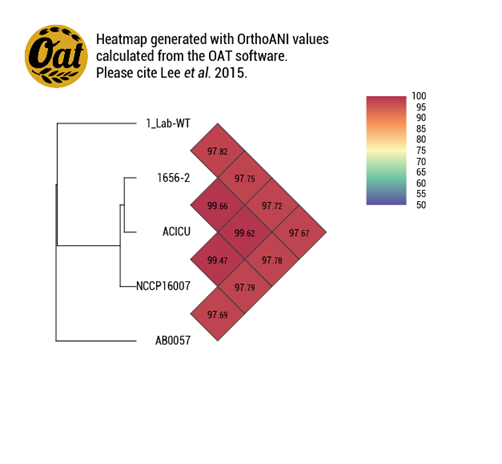
Figure S1. Orthologous average nucleotide identity (OrthoANI) of Lab-WT, NCCP 16007, and other MRAB strains (1656-2, ACICU, and AB0057).


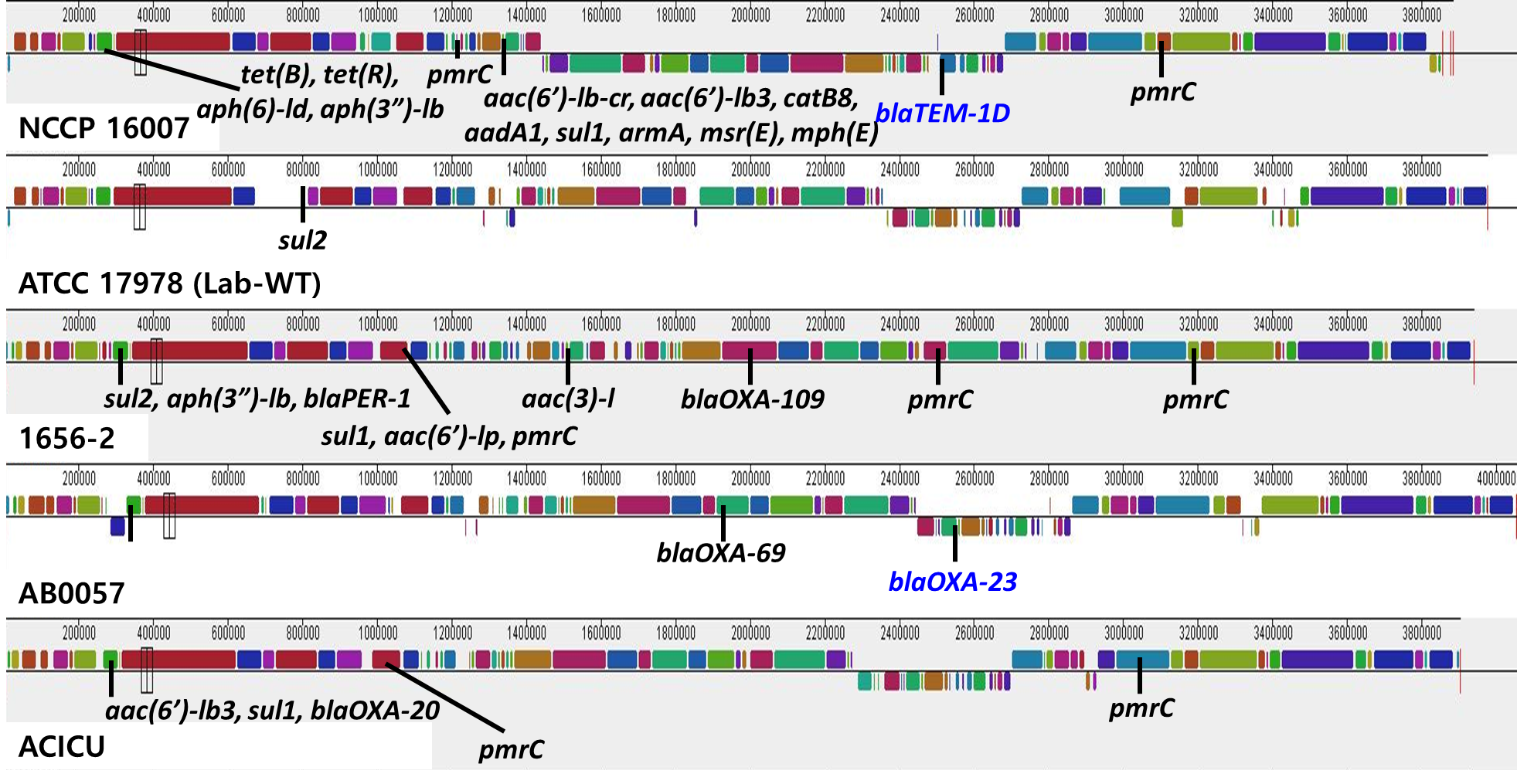
 Figure S2. Genome syntenies in our tested strains (Lab-WT and NCCP 16007 stains), and 13 other well-known clinical isolates. The ARGs of each strain were are accordingly marked. Boxes of the same color refer to theindicate homologous sites in the genomes. The black letters are forward sequences that match the direction of the NCCP 16007 strain in the each strains, and whereas the blue letters are reverse sequences that are in the opposite direction.


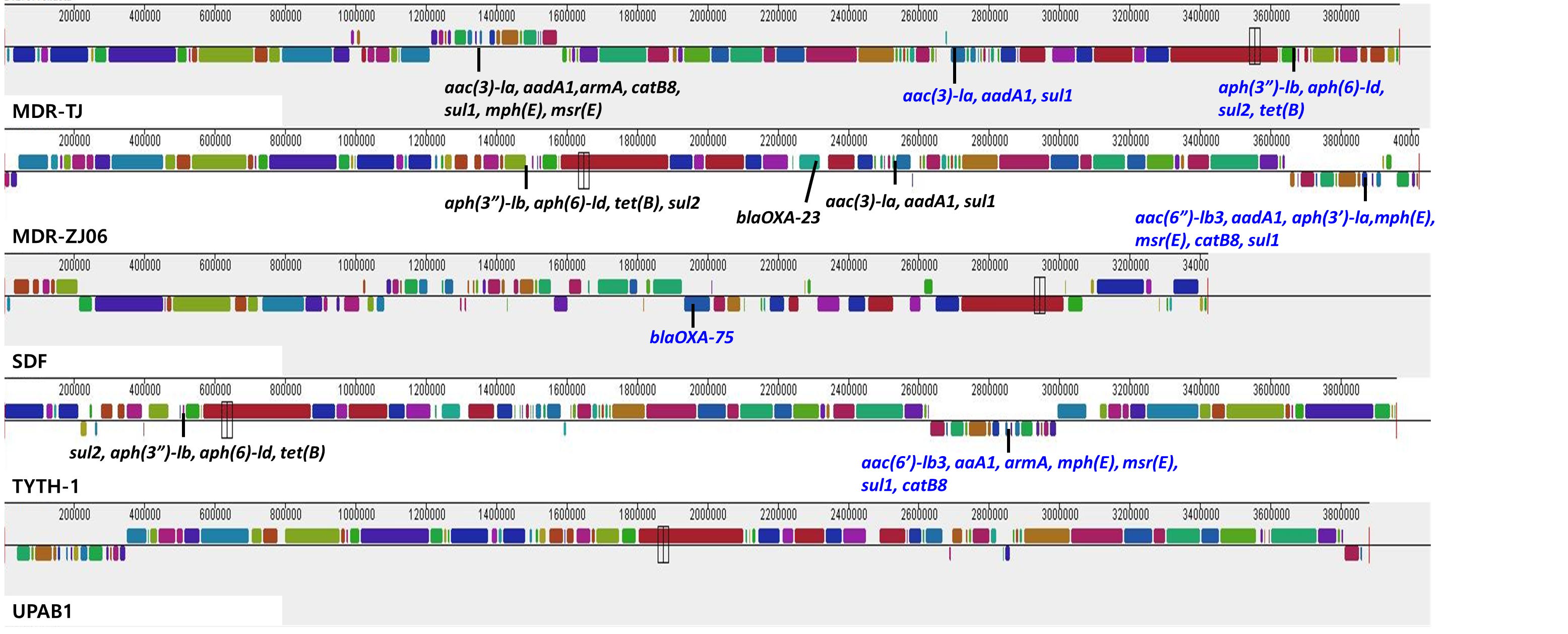


**
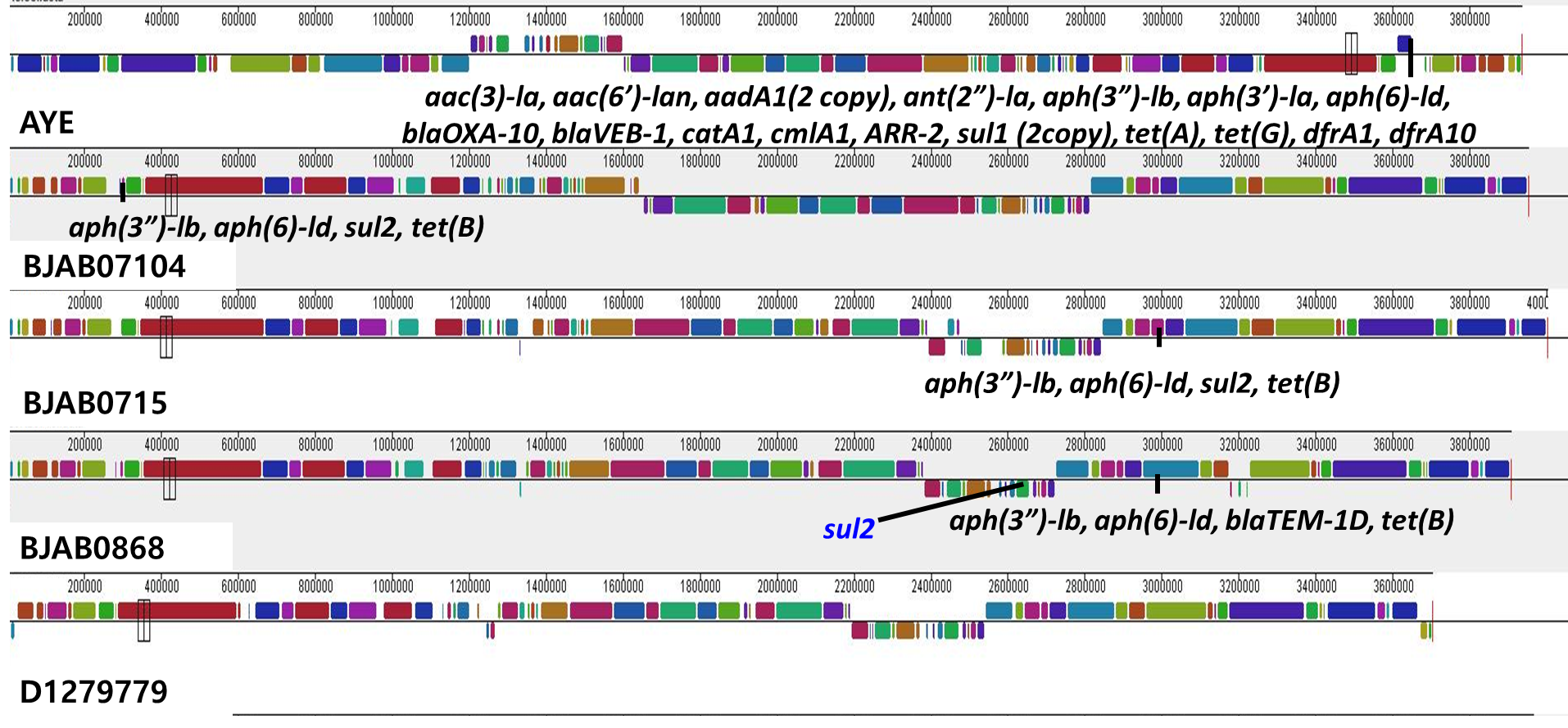
**


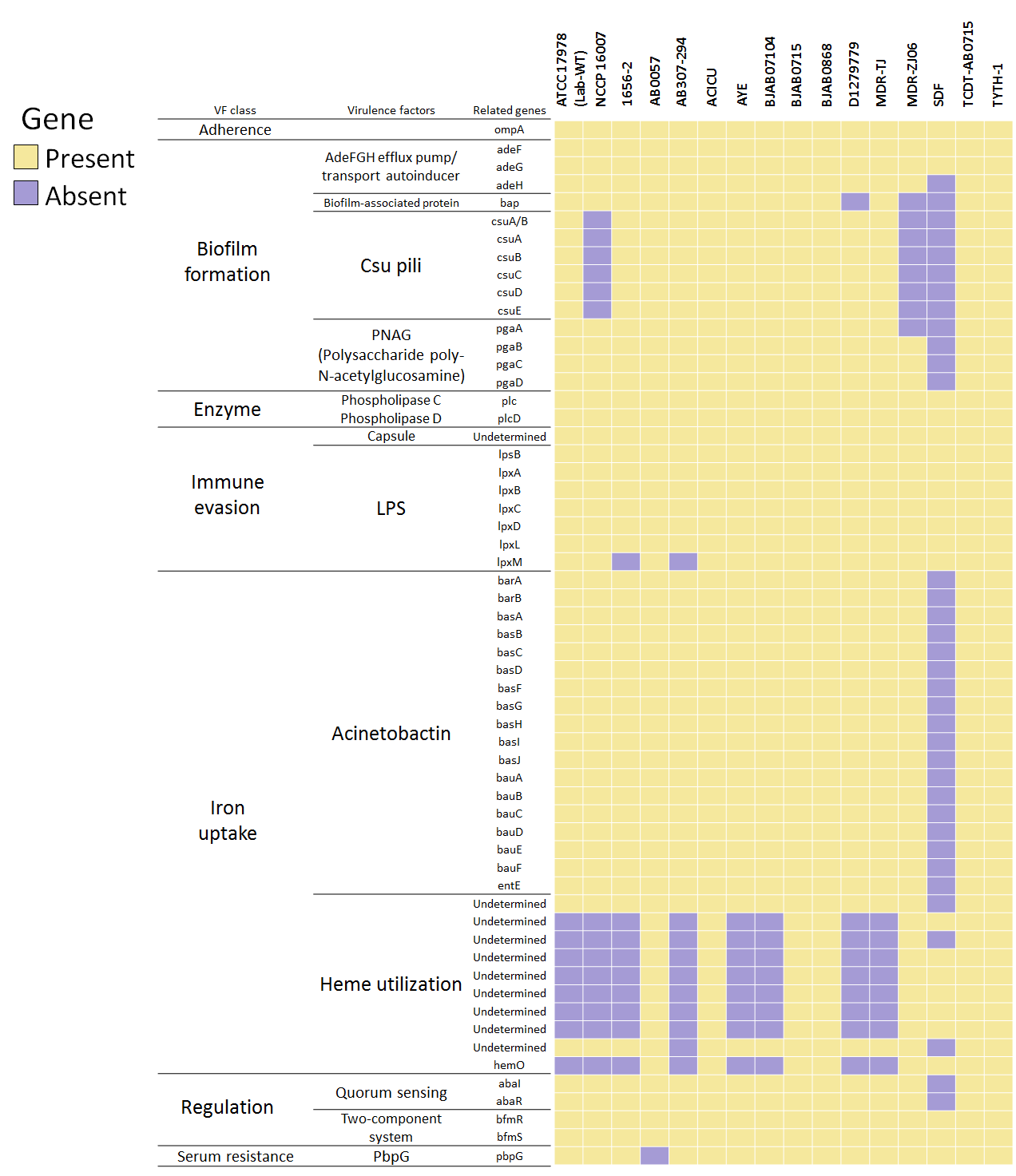
Figure S3. Virulence factors of Lab-WT, NCCP 16007, and other *A. baumannii* strains. The color differencet colors indicates indicate the presence or absence of the virulence-related genes. MRAB strains include 1656-2, AB0057, ACICU, AYE, BJAB07104, BJAB0715, BJAB0868, D1279779, MDR-TJ, MDR-ZJ06, TCDC-AB0715, and TYTH-1. Antibiotic-sensitive isolates are include the *A. baumannii* AB307-0294 and SDF strains.


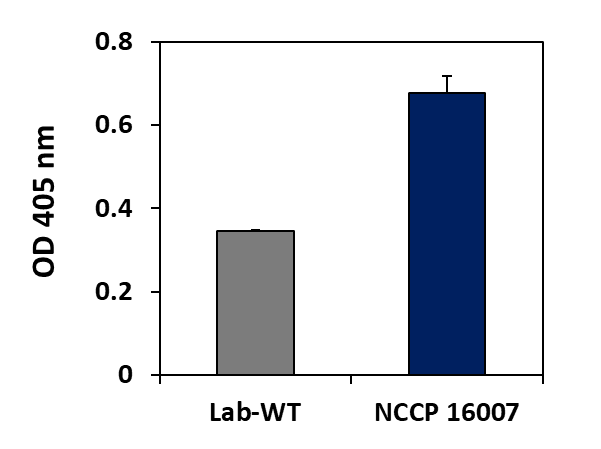
Figure S4. Esterase activity using 4-nitrophenyl acetate of the Lab-WT and NCCP 16007 strains using 4-nitrophenyl acetate.

Table S1. Characteristics and PMB MICs of *A. baumannii* clinical isolates obtained from 2004 to 2013 in South Korea.

| Strain | Year | Region | Source | Genotype | PMB MIC (μg/ml) |
| --- | --- | --- | --- | --- | --- |
| ATCC 17978 Lab-WT (Control) |  |  | Laboratory | OXA-259 | 2 |
| NCCP 16002 | 2013 | Gangwon-do | Sputum | OXA-23, CC 447 | 1 |
| NCCP 12276 | N.A | Busan | N.A | N.A | 2 |
| NCCP 12277 | N.A | Gwangju | Pus | N.A | 1 |
| NCCP 12278 | N.A | Gyeongsangnamdo | Sputum | N.A | 1 |
| NCCP 16000 | 2013 | Gyeonggi-do | Prostate | OXA-23, CC 397 | 1 |
| NCCP 16001 | 2013 | Chungcheongbuk-do | N.A | CC 447 | 1 |
| NCCP 15988 | 2013 | Jeollanam-do | Sputum | OXA-23, ST 191, CC 92 | 2 |
| NCCP 15996 | 2013 | Gyeonggi-do | Urine | OXA-23, ST 451, CC 92 | **256** |
| NCCP 15997 | 2013 | Jeollanam-do | Urine | OXA-23, ST 737, CC 92 | 2 |
| NCCP 15987 | 2013 | Incheon | Pus | ST 359 | 1 |
| NCCP 15990 | 2013 | Gwangju | Sputum | OXA-23, ST 219, CC 92 | 2 |
| NCCP 15991 | 2013 | Kyongsangbuk-do | Urine | OXA-23, ST 357, CC 92 | 2 |
| NCCP 15998 | 2013 | Kyongsangbuk-do | Sputum | ISABA-OXA-51,  ST 229, CC 110 | 2 |
| NCCP 14654 | 2009 | Gyeongsangnamdo | Other | N.A | 2 |
| NCCP 15999 |  |  | Sputum | OXA-23, CC 397 | 4 |
| NCCP 14782 | 2009 | Gyeongsangnamdo | Pus | N.A | 2 |
| NCCP 15989 | 2013 | Jeollabuk-do | Sputum | OXA-23, ST 208, CC 92 | 1 |
| NCCP 16004 | 2013 | Seoul | Urine | ST 1, CC 701 | 1 |
| NCCP 16005 | 2013 | Kyongsangbuk-do | Sputum | ST 1, CC 739 | 2 |
| **NCCP 16007** | 2011 | Seoul | **Urine** | **OXA-23, ST 357, CC 92** | **256** |
| NCCP 16003 | 2013 | Gyeongsangnamdo | Sputum | ST 1, CC 454 | 4 |
| NCCP 15993 | 2013 | Seoul | Pus | OXA-23, ST 368, CC 92 | 1 |
| NCCP 16011 | 2011 | Seoul | N.A | OXA-23, ST 357, CC 92 | 2 |
| NCCP 15994 | 2013 | Kyongsangbuk-do | Pus | OXA-23, ISABA-OXA-51, ST369, CC 92 | 1 |
| NCCP 14655 | 2009 | Gyeongsangnamdo | Pus | N.A | 2 |
| NCCP 14609 |  |  | Pleural fluid | N.A | 1 |
| NCCP 14607 | 2005 | Daegu | Sputum | N.A | 1 |
| NCCP 14606 | 2005 | Daegu | Cerebrospinal fluid | N.A | 2 |
| NCCP 14608 | 2004 | Daegu | Pus | N.A | 2 |
| NCCP 16008 | 2011 | Seoul | N.A | OXA-23, ST 357, CC 92 | 2 |
| NCCP 16009 | 2011 | Seoul | N.A | OXA-23, ST 357, CC 92 | 1 |
| NCCP 15992 | 2013 | Seoul | N.A | OXA-23, ST 358, CC 92 | 1 |
| NCCP 16006 | 2011 | Seoul | N.A | OXA-23, ST 357, CC 92 | 2 |
| NCCP 15995 | 2013 | Seoul | N.A | OXA-23, ST 373, CC 92 | **128** |
| NCCP 16010 | 2011 | Seoul | N.A | OXA-23, ST 357, CC 92 | 2 |
| F-1025 | N.A | N.A | N.A | N.A | 4 |
| F-1208 | N.A | N.A | N.A | N.A | 2 |
| F-1379 | N.A | N.A | N.A | N.A | 2 |
| F-1410 | N.A | N.A | N.A | N.A | 4 |
| F-1629 | N.A | N.A | N.A | N.A | **128** |

N.A: not available

Table S2. Primers used in this study.

| Primer | Sequence (5’-3’) / Description |
| --- | --- |
| *pmrB*_F | GAGGACTGGGCTACCGTTTG / forward primer for PCR and sequencing |
| *pmrB*_R | GCTGGGTCGTTTGGGCAATA / reverse primer for PCR and sequencing |
| *pmrB*_I | ACGCGATTCCGAAGAACTCA / intermediate primer for sequencing |
| *pmrC*_F | AAGTGAGCTCATGCGGTACG / forward primer for qRT-PCR |
| *pmrC*_R | AGTCACTTGGTAACCCGCAC / reverse primer for qRT-PCR |
| b341 | CCTACGGGAGGC/ forward 16s rRNA primer for qRT-PCR |
| 534R | ATTACCGCGGCTGC/ reverse 16s rRNA primer for qRT-PCR |
| *ompA_*F | GATGGCGTAAACCGRGGRACA/ forward primer for qRT-PCR |
| *ompA_*R | CTACAGGAGCAGCAGGCTT/ reverse primer for qRT-PCR |
| *bfmR_*F | CGTGAAGTTCGCCCACACTA/ forward primer for qRT-PCR |
| *bfmR_*R | GAACGGCCACCATTGTCGAT/ reverse primer for qRT-PCR |
| *pilA_*F for NCCP 16007 | AGCTGTTCAATCCGAAATGATG/ forward primer for qRT-PCR |
| *pilA_*R for NCCP 16007 | TGCAGTAAGTACCCAAGTTGAATC/ reverse primer for qRT-PCR |
| *pilA_*F for ATCC 17978 | CTGCTGTTCAATCCGAAATGATG/ forward primer for qRT-PCR |
| *pilA_*R for ATCC 17978 | GCACCCATGTCGAATCGTTTA/ reverse primer for qRT-PCR |

Table S3. Genome features of *A. baumannii* ATCC 17978 and NCCP 16007.

| *A. baumannii* strain | Total size (bp) | | | G+C ratio (%) | | CDS | rRNA | tRNA | Contigs | Accession No. |
| --- | --- | --- | --- | --- | --- | --- | --- | --- | --- | --- |
| ATCC17978 (Origin) | 4,001,457 | Chromosome | 3,976,747 | 38.9 | 39 | 3,370 | 15 | 70 | 3 | CP000521.1 |
|  |  | pAB1 | 13,408 |  | 36 |  |  |  |  | CP000522.1 |
|  |  | pAB2 | 11,302 |  | 35 |  |  |  |  | CP000523.1 |
| NCCP16007 | 3,884,786 | Chromosome | 3,854,994 | 39.0 | 39 | 3,618 | 18 | 73 | 3 | CP049363.1 |
|  |  | pNC1 | 8,031 |  | 35 |  |  |  |  | CP049365.1 |
|  |  | pNC2 | 21,761 |  | 34 |  |  |  |  | CP049364.1 |

Table S4. Source of the IS elements in the NCCP 16007 strain.

| Name | Family | Homolog (%) | Query/Template length (aa) | Position in contig | Product | Source | Accession number |
| --- | --- | --- | --- | --- | --- | --- | --- |
| Tn5393 | Tn3 | 100 | 685/685 | 2489086-2489770 | Resolvase | *Erwinia amylovora* | M96392 |
| IS*Ec29* | IS4 | 100 | 1325/1325 | 1254264-1255588 | Transposase | *Escherichia coli* | FJ187822 |
| IS*Vsa3* | IS91 | 100 | 977/977 | 242469- 243445 | Transposase | *Vibrio salmonicida* | AJ289135 |
| N.A | IS91 | 99 | 512/513 | 1249941-1251482 | Transposase | *Escherichia coli* | WP_098986936 |
| IS*Ec28* | IS5 | 99 | 896/897 | 1251715- 1252611 | Transposase | *Escherichia coli* | FJ187822 |
| IS*Ec63* | Tn3 | 85 | 424/498 | 2494551-2495047 | Transposase | *Escherichia coli* | WP_112017972 |
| IS26 | IS6 | 100 | 820/820 | 2495052-2495871,  2487763-2488582, | Transposase | *Proteus vulgaris* | X00011 |
| IS26 | IS6 | 99 | 818/820 | 1244286-1245105,  1262520- 1263339,  2553347-2554166 | Transposase | *Proteus vulgaris* | X00011 |
| IS15 | IS6 | 100 | 503/503 | 2488583-2489085 | Transposase | *Salmonella panama* | M12900 |
| IntI1 | N.A | 99 | 316/319 | 1244944-1245903 | Class 1 integron integrase | *Klebsiella pneumoniae* | WP_032409260 |
| IS*Aba26* | IS256 | 99 | 1313/1318 | 959027-960344,  3646608-3647925 | Transposase | *Acinetobacter baumannii* | CP007712 |
| IS*Aba24* | IS66 | 100 | 2421/2421 | 1258970-1261391 | Transposase | *Acinetobacter baumannii* | CP006963 |
| IS*Aba1* | IS4 | 100 | 1180/1180 | 3666638-3667817,  3200788-3201967,  2696333- 2697512,  2459102-2460281,  1396785- 1397964,  1829552-1830731,  1785762- 1786941,  1571924-1573103,  2297547- 2298726,  1518915-1520094,  2461862- 2463041,  1169487-1170666, | Transposase | *Acinetobacter baumannii* | AY758396 |
| IS*Aba1* | IS4 | 99 | 1175/1180 | 1523004- 1524183,  236542- 237721,  232917- 234096 | Transposase | *Acinetobacter baumannii* | AY758396 |
| IS*Alw4* | IS3 | 95 | 1250/1309 | 1012159-1013467,  96358-97666 | Transposase | *Acinetobacter lwoffii* | KX426227 |
| IS*Aba40* | IS5 | 99 | 1038/1039 | 94695-95733,  411610- 412648 | Transposase | *Acinetobacter baumannii* | N.A |
| IS*Aba40* | IS5 | 82 | 598/731 | 92066-92796,  108652-109382 | Transposase | *Acinetobacter baumannii* | N.A |
| IS*Alw23* | IS3 | 93 | 1213/1309 | 1012159-1013467,  96358- 97666 | Transposase | *Acinetobacter lwoffii* | N.A |
| IS*Aba2* | IS3 | 91 | 1192/1310 | 1012159-1013467,  96358- 97666 | Transposase | *Acinetobacter baumannii* | AY665723 |
| IS*Alw5* | IS3 | 90 | 1183/1309 | 1012159- 1013467,  96358- 97666 | Transposase | *Acinetobacter lwoffii* | N.A |

N.A: not available

| Antibiotics | Resistance gene | Identify (%) | Query/Template length (bp) | Position in contig | Product | Source | Accession number | GC ratio  of gene (%) | GC ratio  of source (%) |
| --- | --- | --- | --- | --- | --- | --- | --- | --- | --- |
| β-lactam | *blaADC-25* | 100 | 1152 / 1152 | 2740797-2741948 | β -lactamase | *Acinetobacter baumannii* strain 17368 | EF016355 | 36 | 39 |
|  | *blaOXA-259* | 100 | 825 / 825 | 1765468-1766292 | β -lactamase | *Acinetobacter* *baumannii* strain 1979/W51 | KF057028 | 39 | 39 |
| Sulphonamide | *sul2* | 100 | 816/816 | 798900-799715 | Dihydropteroate synthase | *Vibrio* *cholerae* strain MO10 SXT | AY034138 | 61 | 47.5 |

Table S5. Sources of AR genes in *A. baumannii* ATCC 17978.
